# Divergence of the bZIP Gene Family in Strawberry, Peach, and Apple Suggests Multiple Modes of Gene Evolution after Duplication

**DOI:** 10.1155/2015/536943

**Published:** 2015-12-07

**Authors:** Xiao-Long Wang, Yan Zhong, Zong-Ming Cheng, Jin-Song Xiong

**Affiliations:** College of Horticulture, Nanjing Agricultural University, Nanjing 210095, China

## Abstract

The basic leucine zipper (bZIP) transcription factors are the most diverse members of dimerizing transcription factors. In the present study, 50, 116, and 47* bZIP* genes were identified in* Malus domestica* (apple),* Prunus persica* (peach), and* Fragaria vesca* (strawberry), respectively. Species-specific duplication was the main contributor to the large number of* bZIPs* observed in apple. After WGD in apple genome, orthologous* bZIP* genes corresponding to strawberry on duplicated regions in apple genome were retained. However, in peach ancestor, these syntenic regions were quickly lost or deleted. Maybe the positive selection contributed to the expansion of clade S to adapt to the development and environment stresses. In addition, purifying selection was mainly responsible for* bZIP* sequence-specific DNA binding. The analysis of orthologous pairs between chromosomes indicates that these orthologs derived from one gene duplication located on one of the nine ancient chromosomes in the Rosaceae. The comparative analysis of* bZIP* genes in three species provides information on the evolutionary fate of* bZIP* genes in apple and peach after they diverged from strawberry.

## 1. Introduction

Many of the biological processes in cell or organism, such as responses to the environment and progression through the cell cycle, metabolic and physiological balance are influenced or controlled by regulation of gene expression at the level of transcription. Development is based on the cellular capacity for differential gene expression and is controlled by transcription factors acting as switches of regulatory cascades [1]. Alterations in the expression of genes coding for transcription factors (TFs) are emerging as a major source of the diversity and change that underlie evolution [2]. Presently, at least 64 families of transcription factors have been identified in the plant kingdom [3]. The bZIP proteins represent a large family of TFs with a DNA-binding domain rich in basic amino acid residues, which is adjacent to a leucine zipper dimerization domain (N-x7-R/K-x9) for sequence-specific DNA binding, and a leucine zipper, which is composed of several heptad repeats of Leu or other bulky hydrophobic amino acids, such as Ile, Val, Phe, or Met, for dimerization specificity [4–7]. In addition, the majority of characterized plant* bZIP* genes to date have been associated with enhancing plant tolerance to diverse types of abiotic stress [8–14].

Recent* bZIP* gene sequence analyses in* Arabidopsis* [5], rice [6], castor bean [15], maize [16], sorghum [17], cucumber [18], and grape [19], further indicated illegitimate recombination (IR) as a major source of duplications and deletions [20]. The evidence obtained from these analyses suggests that gene duplications in a common ancestor of those plants gave rise to* bZIP* genes. Therefore, the very earliest origins of the* bZIP* gene family are associated with a series of gene duplications. A total of 75 and 89* bZIP* genes have been identified in* Arabidopsis* [5] and rice (*Oryza sativa*) [6], respectively. The* bZIP* genes in these two genera have been classified into 10 groups and 11 groups, respectively, based on DNA binding specificity and sequence similarity.

The Rosaceae is one of the most economical plant families [21] composed by some 90 genera with over 3000 distinct species which have *x* = 7 to *x* = 17 chromosomes [22]. According to a phylogenetic treatment based on DNA sequence, data of nuclear and chloroplast genomic regions in Rosaceae reclassified the genus into Dryadoideae, Rosoideae, and Spiraeoideae, each containing a number of distinct supertribes [22].* Prunus* and* Malus* are included in the Spiraeoideae, supertribe Amygdaleae, and Pyrodae (tribe Pyrinae), respectively, whilst* Fragaria* is included in the Rosoideae, supertribe Rosodae (tribe Fragariinae) [23]. After the rapid evolution of Rosaceae, members of the family display remarkable phenotypic diversity, plant habit, chromosome number, and fruit type which evolved independently on more than one opportunity [24, 25]. A better understanding of how the* bZIP* genes within the Rosaceae arose would provide an insight into how evolution can lead rapidly to diversification. The genomes of three Rosaceous species, woodland strawberry [26], domesticated apple [27], and peach [28], have been recently sequenced, providing an opportunity to conduct a high-resolution comparison of their genomes. In this study, we identified 50, 116, and 47 bZIP transcription factors based on the complete genome sequences of strawberry, apple, and peach. Further, through phylogenetic analysis,* Ka/Ks* ratios of genes and bZIP domains, and orthologous relationships among chromosomes, we explain the evolutionary history of* bZIP* genes in detail.

## 2. Methods

### 2.1. Data Resources and the Identification of* bZIP* Genes

*Fragaria vesca* (strawberry, v1.1),* Malus domestica* (apple, v1.0), and* Prunus persica* (peach, v1.0) genomic and annotation data were downloaded from the Genome Database for Rosaceae (GDR, http://www.Rosaceae.org/) [26–28]. The genome sequences of* Brassica rapa* (v1.3),* Solanum lycopersicum* (iTAG2.3),* Chlamydomonas reinhardtii* (v5.5),* Theobroma cacao* (v1.1),* Selaginella moellendorffii* (v1.0),* Populus trichocarpa* (v3.0),* Medicago truncatula* (Mt4.0v1),* Cucumis sativus* (v1.0),* Carica papaya* (ASGPBv0.4), and* Physcomitrella patens* (v3.0) were downloaded from Phytozome (http://www.phytozome.net/) [29]. Genomic data on* M. acuminate* (v1) (http://banana-genome.cirad.fr/),* Saccharomyces cerevisiae* (v1) (http://www.yeastgenome.org/), and* Cyanidioschyzon merolae* (http://merolae.biol.s.u-tokyo.ac.jp/) were also downloaded for inclusion in the analyses. The* bZIP* genes in the genomes of* Vitis vinifera* [19],* Arabidopsis thaliana* [5], and rice (*Oryza sativa*) [6] were previously identified.

The Hidden Markov Model (HMM) profiles of the bZIP domain (PF00170) were retrieved from Pfam 27.0 [30] and used for identifying the* bZIP* genes from the downloaded database of genomes using HMMER3.0 [31]. All output genes with a default *E*-value (<1.0) were collected and the online software SMART (http://smart.embl-heidelberg.de/) was used to confirm the integrity of the bZIP domain using an *E*-value of <0.1 [32]. Incorrectly predicted genes were removed. Finally, the sequences of nonredundant genes with high confidence were collected and assigned as* bZIP* genes.

### 2.2. Alignment and Phylogenetic Analysis of* bZIP* Genes

Based on the location (Table S2 in Supplementary Material available online at http://dx.doi.org/10.1155/2015/536943) predicted in the Pfam 27.0 [30] of conserved domains in complete predicted bZIP protein sequences, the conserved domain sequences of bZIP proteins were extracted and aligned using ClustalX (version 1.83) [33]. The phylogenetic trees were generated with MEGA 5.0 [34] using the Neighbor-Joining (NJ) method and number of difference model [35]. 1,000 bootstraps were used to evaluate the significance of the phylogenetic trees.

### 2.3. Synteny Analysis of Strawberry, Apple, and Peach Genomes

For synteny analysis, syntenic genes within the strawberry, apple, and peach genomes, as well as between strawberry and apple, strawberry and peach, peach and apple genomes, were downloaded from the Plant Genome Duplication Database [36] (PGDD, http://chibba.agtec.uga.edu/duplication/) and those containing* bZIP* genes were identified and analyzed. We identified the syntenic gene pairs from the same and different species within the same clade from phylogenetic analysis as paralogous and orthologous genes.

### 2.4. Estimation of Nonsynonymous Substitutions and Synonymous Substitutions

The nucleotide sequences of* bZIP* gene and bZIP domain in each clade except for UN were aligned by using Clustalw 2.0 [37]. The nonsynonymous substitutions (*Ka*) and synonymous substitutions (*Ks*) and nonsynonymous to synonymous substitution ratios (*Ka*/*Ks*) were estimated in each gene family according to the alignments in MEGA 5.0 [38]. In order to detect selection pressure of different clades of* bZIPs* in phylogenetic tree (A, B, C, D, E, F, G, H, I, and S),* Ka/Ks* ratio greater than 1, less than 1, and equal to 1 represents positive selection, negative or stabilizing selection, and neutral selection, respectively. The software in SPSS version 19.0 (SPSS, Chicago, IL, USA) was used for statistical analysis. The statistical significance of* Ka/Ks* was defined based on Duncan's multiple range test and *P* value of < 0.05 as statistically significant.

## 3. Results

### 3.1. Identification and Comparative Analyses of* bZIP* Genes in Nineteen Species

**Figure 1:**
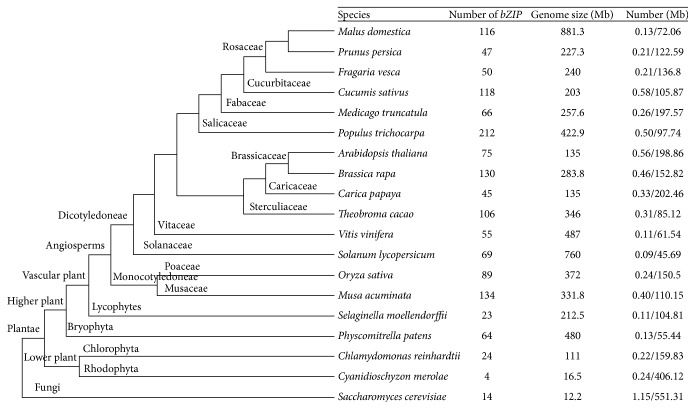
Phylogenetic relationships, number of* bZIP* genes, genome size, bZIP density, and overall gene density of the nineteen species analyzed. The bZIP density and overall gene density of the nineteen species analyzed were separated by parenthesis. The bZIP density was followed by overall gene density.

The sequences of 1441* bZIP* sequences in 19 genomes, ranging from fungi to Plantae, including the three Rosaceous species, were used to analyze the evolution of this gene family (Figure 1 and Table S1). In the genome assemblies of strawberry, apple, and peach, 50, 116, and 47* bZIP* genes were identified, respectively, using the HMM profile from the Pfam database [39] (Table S2). The number of* bZIP* genes varies from 4 (*C. merolae*) to 212 (*P. trichocarpa*) in 19 species with the genome size from 12.2 Mb (*S. cerevisiae*) to 881.3 Mb (*M. domestica*). Furthermore, we found that the number of* bZIP* genes in six higher plant species was more than 100.

The total number of* bZIP* genes in strawberry and peach was very similar. However, it is important to note that the number of* bZIP* genes in these two species was much less than the number in apple (116). The number of* bZIP* genes in strawberry (50) and peach (47) was also much smaller than the number in most of the 19 species (Figure 1), which may infer that only a small number of gene duplication events contributed to the* bZIP* members in these two species. The number of apple* bZIP* genes (116) was similar to the number in* C. sativus* (118) but less than the number found in* P. trichocarpa* (212),* B. rapa* (130), and* M. acuminate* (134) (Figure 1). In contrast, the density of* bZIP* genes in the three Rosaceous species was very distinct and not related to the number of* bZIP* genes present. The* bZIP* density in the apple genome (0.13) was lower than that in strawberry (0.21) and peach (0.21) and only exceeded the density observed in* V. vinifera* (0.11),* S. moellendorffii* (0.11), and* S. lycopersicum* (0.09) (Figure 1). By contrast, the apple genome also had a lower overall gene density (72.06), which is probably the reason for low* bZIP* density in the apple genome.

### 3.2. Phylogenetic Analysis of* bZIP* Genes in Three Rosaceous Species

A phylogenetic analysis was performed for the* bZIP* genes in the three Rosaceous species using the bZIP domains in strawberry, apple, and peach, as well as* Arabidopsis* [5], in order to further elucidate the evolution of this gene family (Figure S1). Since the* bZIP* genes of* Arabidopsis* have already been clustered, we were able to compare the clustering of the* bZIP* genes of Rosaceous species with the clustering from* Arabidopsis*. Surprisingly,* AtbZIP31*,* AtbZIP33*, and* AtbZIP74* were different from other* bZIP* genes in that they formed individual clades containing only* bZIP* genes of* Arabidopsis*, suggesting that these individual clades may be specific to* Arabidopsis* (Figure S1).

**Figure 2:**
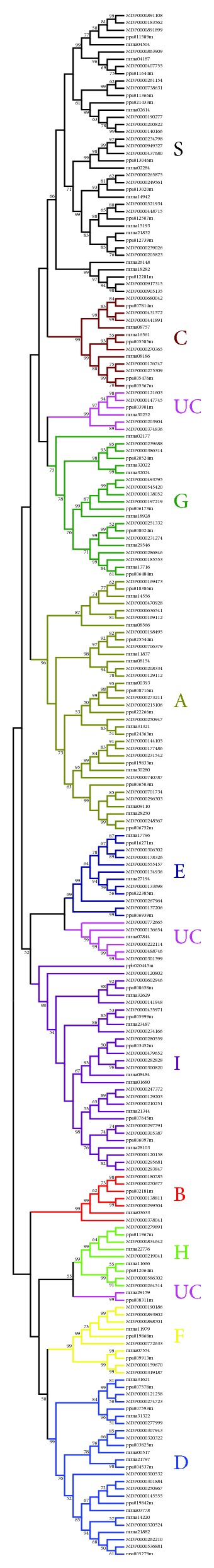
Phylogenetic analysis of* bZIP* members in strawberry, apple, and peach. Phylogenetic analysis of bZIP proteins in strawberry (mrna), apple (MDP), and peach (ppa). Only bootstrap values larger than 50% are indicated. Different colors can be used to distinguish the different subgroups. The names of each subgroup are listed on the right. UC represented “unique clades.”

The results indicated that the ten clades (A, B, C, D, E, F, G, H, I, and S) obtained in our phylogenetic tree were in agreement with the clustering and classification of* bZIP* genes in Arabidopsis [5] (Figure 2). However, a few genes formed three small unique clades (UC, Figure 2) in the phylogenetic tree produced from our analyses. This observation supports the hypothesis that these three unique clades may have had independent evolutionary trajectories from the other clades.

All of the clades from Figure 2 include genes from all of the three species. The number of strawberry, apple, and peach* bZIP* genes, respectively, in each of the clades were A (9, 18, 8); B (1, 5, 1); C (3, 6, 4); D (7, 12, 6); E (2, 8, 3); F (2, 6, 2); G (6, 10, 4); H (2, 5, 2); I (6, 17, 6); and S (9, 21, 9). Moreover, the phylogenetic tree of the three Rosaceous species indicated that the* bZIP* genes in strawberry and peach have few paralogs with “one-to-one” topology (two paralogs clustered together in a clade), suggesting that most of them were generated before speciation of strawberry. In contrast, there were many clades with “one-to-one” or “one-to-many” topologies (more than two paralogs clustered together in a clade) in apple, indicating that species-specific duplication events contributed greatly to the large number of apple* bZIPs*.

### 3.3. Nonsynonymous and Synonymous Substitution of* bZIP* Genes

**Figure 3:**
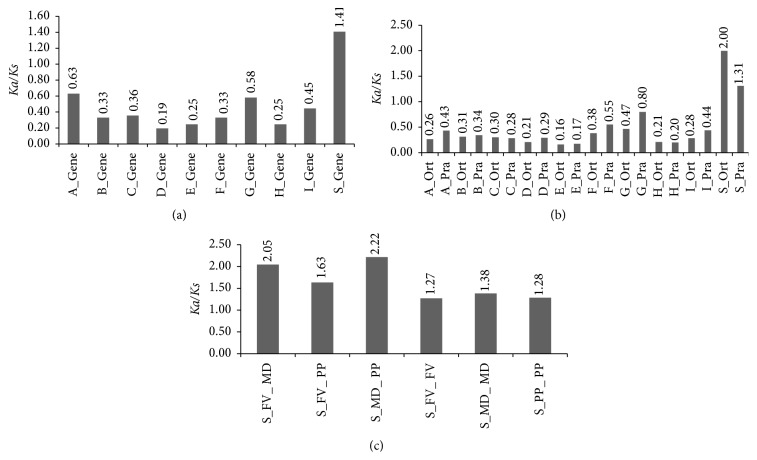
*Ka/Ks* ratios of* bZIP* genes. (a)* Ka/Ks* ratios of genes in clades A–S. (b)* Ka/Ks* ratios of paralogous and orthologous gene pairs in clades A–S. (c)* Ka/Ks* ratios of paralogs (FV_FV, MD_MD, and PP_PP) and orthologs (FV_MD, FV_PP, and MD_PP) in clade S. The* Ka/Ks* ratios are located in the top of the graph.

Our result indicates that most clades (A, B, C, D, E, F, G, H, and I) had* Ka/Ks* ratios less than 1 (Figure 3(a)), demonstrating that most genes of those clades were undergoing a purifying selection in the three species. Among all the gene pairs in the clades, 25 (7.99% of clade A), 1 (2.13% of clade E), 16 (9.58% of clade G), and 12 (5.33% of clade I) pairs had* Ka/Ks* ratio approximately equal to 1 (*Ka/Ks* ratio = 0.8~1.0) for* bZIP* genes in strawberry, apple, and peach (Table S3). However, 52 (16.61% of clade A), 15 (8.98% of clade G), 1 (4.55% of clade H), and 15 (6.67% of clade I) gene pairs had* Ka/Ks* ratios greater than 1 for* bZIP* genes (Table S3), which indicates that some of* bZIP* genes were under positive selection or relaxed selection for gene pairs with* Ka*/*Ks* approximately equal to 1. It is worth noting that* Ka/Ks* ratio of gene pairs in clade S is significantly greater than other clades (*P* < 0.05), which illustrated that* bZIP* genes were under strongly positive selection (Figure 3(a)).

In order to explain* Ka/Ks* ratio distribution of gene pairs in each clade, we compared* Ka/Ks* ratio of the orthologous and paralogous gene pairs in strawberry, apple, and peach (Table S4). It is indicated that the* Ka/Ks* ratio of paralogs is bigger than orthologs in each clade except for clades C, H, and S (Figure 3(b)). Most of orthologs and paralogs exhibit a low level* Ka/Ks* ratio (*Ka/Ks* ratio = 0.16~0.80) in different clades (A, B, C, D, E, F, G, H, and I) analyzed (Figure 3(b)). However, the ones of orthologs (*Ka/Ks* ratio = 2.00) and paralogs (*Ka*/*Ks* ratio = 1.31) in clade S are obviously greater than 1 and significantly higher than orthologs and paralogs in other clades (*P* < 0.05). Orthologs and paralogs in clade S could be further divided into three subgroups separately, FV_PP (between strawberry and peach)/MD_FV (between apple and strawberry)/MD_PP (apple and peach) and FV_FV (within strawberry)/MD_MD (within apple)/PP_PP (within peach). Orthologs in the MD_PP have a highest* Ka/Ks* ratio (2.22) and paralogs in the FV_FV have a lowest* Ka/Ks* ratio (1.27) (Figure 3(c)).

### 3.4. Nonsynonymous and Synonymous Substitution of bZIP Domains

**Figure 4:**
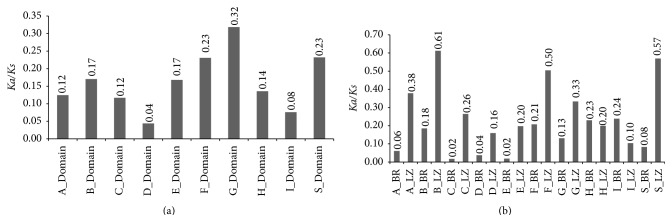
*Ka/Ks* ratios of* bZIP* domains. (a)* Ka/Ks* ratios of domains in clades A–S. (b)* Ka/Ks* ratios of BR and LZ domains in clades A–S. The* Ka/Ks* ratios are located in the top of the graph.

For getting a more in-depth exploration in selection pressure of* bZIP* genes in different clades during their evolution, we compared the* Ka/Ks* ratio of bZIP domains in each clade (Table S5). We found that all clades with* Ka/Ks* ratios ranging from 0.04 (clade D) to 0.32 (clade G) were less than 0.4 (Figure 4(a)). It is suggested that a strong negative selection plays the leading roles in the evolution of bZIP domains.

Basic leucine zipper (bZIP) proteins, one of the largest families of transcription factors in plants, are characterized by a basic region (BR) responsible for sequence-specific DNA binding, an adjacent heptad leucine repeat, and the leucine zipper (LZ) [40]. It is concluded that all BR domains ranging from 0.02 (BR of clade C) to 0.24 (BR of clade I) and LZ domains ranging from 0.1 (LZ of clade I) to 0.61 (LZ of clade B) were undergoing negative selection (Figure 4(b),Table S5). Interestingly,* Ka/Ks* ratio of BR domain is less than the ones of LZ domain in each clade except for clades H and I (Figure 4(b)).

### 3.5. Evaluation of Orthologous* bZIP* Genes between Strawberry, Apple, and Peach

**Figure 5:**
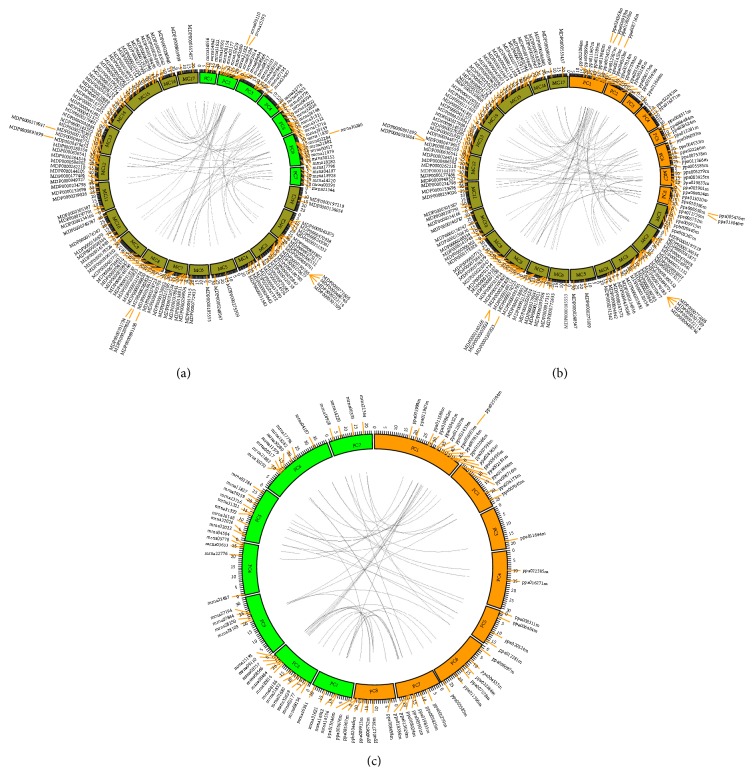
Evaluation of orthologous* bZIP* genes between strawberry, apple, and peach. (a) Seven strawberry (FC1 to FC7) and seventeen apple chromosome (MC1 to MC17) maps are based on the orthologous pair position and demonstrate a highly conserved syntenic relationship. (b) Seventeen apple chromosome (MC1 to MC17) and peach (PC1 to PC8) maps are based on the orthologous pair positions and demonstrate highly conserved synteny. (c) Seven strawberry (FC1 to FC7) and peach (PC1 to PC8) maps are based on the orthologous pair positions, and demonstrate highly conserved synteny.

In order to trace the evolutionary history of* bZIP* genes among the three Rosaceous species, orthologous regions of* bZIP* genes in the three Rosaceous species were subjected to a comparative analysis in order to ascertain the evolutionary history of* bZIP* genes in the Rosaceae. Using Circos software [41], 57 orthologous gene pairs were identified between strawberry and apple (FV_MD) (Figure 5(a)), 64 between apple and peach (MD_PP) (Figure 5(b)), and 50 between strawberry and peach (FV_PP) (Figure 5(c)). Collectively, these data are presented in Table S6 and Figure 5.

Out of the 57 gene pairs present in the strawberry and apple genomes (Figure 5(a)), 20 strawberry* bZIP* genes correspond to one copy (Type 1), 17 genes correspond to two copies (Type 2), and one gene corresponds to three copies (Type 3) in apple. Therefore, 56* bZIP* genes in the apple genome have 38 corresponding genes in the strawberry genome. In all three types, some genes have preserved and exhibit the same number of exons (Table S6). Out of 50 gene pairs present in the strawberry and peach genomes (Figure 5(c)), 26 strawberry* bZIP* genes correspond to one copy (Type 1), 9 genes to two copies (Type 2), and 2 genes to three copies (Type 3) in peach. Collectively, 37 strawberry* bZIP* genes corresponded to 38* bZIP* genes in the peach genome. Genes of all three types in strawberry and peach have preserved similar exon configurations (Table S6). Based on the 30 overlapping* bZIP* strawberry genes, the data collectively indicate that 45* bZIP* genes, representing 90% of the total number of* bZIP* genes in the strawberry genome, were ancestral and underwent different duplication events after the divergent speciation of apple and peach. Additionally, 56* bZIP* genes, representing 48.3% of the total number of* bZIP* genes in the apple genome, were retained on duplicated regions. In addition, 38* bZIP* genes, representing 80.9% of the total number of* bZIP* genes in the peach genome, were retained on syntenic blocks. These data further indicate that most of the* bZIP* genes in strawberry and peach experienced a low level of duplication events compared to the number of duplication events in the apple genome. These findings are consistent with the results of a previous study which reported that a recent whole genome duplication (WGD) event occurred in apple 60–65 million years ago [27].

### 3.6. Orthologous Relationships among Chromosomes

In order to understand the influence of the WGD in apple on the* bZIP* gene family in the Rosaceae, the major distribution of orthologous chromosomes was identified and compared between paired combinations of strawberry, apple, and peach according to the classification reported by Jung et al. [42] (Table S6, Table S7). The orthologous relationship between chromosomes of peach and strawberry made it evident that the majority of* bZIP* genes on peach chromosomes PC2, PC3, PC5, and PC8 were located on a single homologous FC7, FC6, FC5, and FC2 chromosome in strawberry, respectively. The majority of genes on PC6 and PC7 were also located on strawberry chromosomes, FC1 and FC6. Additionally, 35.71% of the* bZIP* genes on strawberry chromosome FC2 had an orthologous relationship to the PC1. Both ppa016271m and ppa022385m of PC4, however, were located on the FC6 chromosome of strawberry.

The relationship between peach and apple at the chromosome level was more complex than the relationship between peach and strawberry. 66.67%, 66.67%, 50%, 50%, and 50% of* bZIP* genes on five apple chromosomes sets, MC2/MC7, MC9/MC17, MC3/MC11, MC14/MC6, and MC2/MC15, respectively, have their orthologous genes corresponding to the chromosomes PC2, PC3, PC4, PC5, and PC7 of peach. Orthologous genes on PC6 corresponded to major genes on four apple chromosomes, MC3, MC4, MC11, and MC12 (Table S6, Table S7).

## 4. Discussion

### 4.1. Evolutionary History of* bZIP* Family in Three Species of the Rosaceae

The bZIP transcription factor family is one of the largest and most diverse families of transcriptional regulators in eukaryotic organisms [15]. In the present study, the bZIP transcription factor family in 16 species, including 13 higher plants, 2 lower plants, and one fungus, was analyzed, in an effort to better understand the evolution of this gene family in the Rosaceae. It has been suggested that the* bZIP* gene family existed before the divergence of higher and lower plant species, even in the fungi, which consists with foundation in Wang et al. (2011) research [17]. An uneven distribution of* bZIP* copies among the 19 species was identified, suggesting that the* bZIP* genes within each species had undergone different levels of gene duplication with larger expansion after the divergence of higher and lower plants. For example, the numbers of copies of* bZIP* genes were as follows:* O. sativa* (89),* Cucumis sativus* (118), and* Populus trichocarpa* (212). These observations suggest that specific functional expansion may have resulted from environmental selection pressure or specialization in processes of growth and development, including stress responses [14, 43–46] and abscisic acid (ABA) signaling [10, 11, 47]. As a result of evolutionary pressure and/or environmental selection, critical genes or components of genes were retained, whereas others were deleted or lost [48].

We identified 50 and 47* bZIP* genes in the genomes of strawberry and peach, respectively. This number is similar with those of previous genome-wide studies on some other species, indicating the presence of 64* bZIP* homologs in cucumber [18], 55 in grapevine [19], and 49 in castor bean [15]. The* bZIP* homologs in apple (116) were consistent with the numbers in maize (120) [16] and sorghum (92) [17].* bZIP* genes in strawberry and peach are much lower than that in apple which has a much larger genome size. These observations support the hypothesis that the WGD [27] event which occurred in apple resulted in significant amplification in the number of apple* bZIP* genes. On the other hand, a low level of gene duplication events may have contributed to the number of* bZIP* genes in strawberry and peach.

### 4.2. *bZIP* Genes Expansion in the Rosaceae

The phylogenetic tree of the* bZIP* gene family generated in this study for Rosaceous species is supported by Liu et al. [19], Nijhawan et al. [6], and Wei et al. [16]. Each of the clades included at least 7* bZIP* genes from the 39* bZIP* genes identified in the 3 species examined, indicating that many of the* bZIP* genes originated through a process of gene duplication. The widespread existence of paralogs and orthologs with “one-to-one” or “one-to-many” topologies in the Rosaceous species examined suggests that species-specific duplication was the main contributor to the large number of* bZIPs* observed in apple. The number of* bZIP* genes in each of the three species was highly variable, indicating that most of the gene duplication events occurred after evolutionary divergence of each lineage. It is also likely that both WGD and a series of rearrangements occurred during the evolution of certain species.

Extensive genome and EST sequencing of plant species has revealed a substantial history of WGD events [49, 50]. In the Rosaceae, an evolutionary trend toward fruit development and specialization may have been partially based on gene duplication. For example, WGD in apple has resulted in the creation of large families of paralogous genes [27]. In our analysis of the apple genome, 56 (48.3%)* bZIP* genes were retained on duplicated regions. Therefore, the involvement of WGD in the expansion of the* bZIP* gene family in apple is quite evident. Polyploidy provides an excellent genomic resource to study retention and loss of multicopy genes [48, 51]. Following WGD, genes can suffer a variety of fates ranging from massive gene loss to the development of a central role in an essential aspect of the plant [52]. A comparative analysis of* bZIP* genes in strawberry, apple, and peach led us to hypothesize that, after WGD in an apple ancestor, orthologous* bZIP* genes corresponding to strawberry on duplicated regions in apple genome were retained. On the other hand, in the peach ancestor, these syntenic regions were quickly lost or deleted, perhaps due to issues associated with an imbalance in gene dosage [53, 54].

### 4.3. Selection Pressure of* bZIP* Genes and bZIP Domains in All Clades

Furthermore,* Ka/Ks* ratios were estimated to detect the diversifying selection pressure on different clades (except for UN clades). The results showed that the* Ka/Ks* ratios for gene pairs in nine clades (A, B, C, D, E, F, G, H, and I) were <1, with most of them being even less than 0.6, suggesting strong purifying selection (Figure 3(a)). However, the other pairs in clade S seemed to be under positive selection, as their* Ka/Ks* ratios were >1. Also, in the phylogenic tree of Rosaceae, we found the biggest clade (S) containing 39 genes (21, 9, and 9 for apple, peach, and strawberry, resp.). Much interest focuses on positive selection (adaptive molecular evolution) associated with adaptation and evolution of new forms or functions in that nonsynonymous mutations offer fitness advantages to the protein [55, 56]. Zhao et al. have concluded that functional gain and divergence of transcription factors were driven by distinct positive selection on their transcription activation domains [57]. Based on the derivative data from monocot and dicot species imply that homologues of S bZIPs are also transcriptionally activated after stress treatment [58], such as drought, cold, and wounding, or are specifically expressed in defined parts of the flower [59, 60]. The positive selection may have contributed to the expansion of clade S to adapt to the development and environment stresses.

The bZIP transcription factors contain a highly conserved bZIP domain composed of two structural features: a basic region (N-X_7_-R/K-X_9_) for sequence-specific DNA binding and a leucine zipper composed of several heptad repeats of Leu or other bulky hydrophobic amino acids, such as Ile, Val, Phe, or Met, for dimerization specificity [5–7]. Additionally,* bZIP* domains of all clades also appeared as stronger purifying selection. A purifying selection may aid in the detection of regions or residues of functional importance [55]. These results suggested that functions of genes in major clades did not diverge much along with the genome evolution after the duplication events. Possibly because of the rapid evolution, members of the Rosaceae display remarkable phenotypic diversity, with common morphological synapomorphies not readily identifiable [23]. It is worth noting that paralogs were undergoing stronger purifying selection than orthologs in each clade except for clades C, H, and S (Figure 3(b)), which probably accelerates the process of morphological diversity, plant habit, and fruit type within the Rosaceae. From Figure 4(b), we conclude that BR domains were under stronger purifying selection than LZ domains in each clade except for clades H and I, suggesting that purifying selection was mainly responsible for* bZIP* sequence-specific DNA binding.

### 4.4. Orthologous Pairs between Chromosomes

Peach, at both the macro- and microsyntenic levels, has the most conserved karyotype in relation to the ancestral genome configuration for the Rosaceae [42]. Dirlewanger et al. [61] compared* Malus* and* Prunus* and found strong evidence that single linkage groups in the diploid* Prunus* were homologous to two distinct homologous linkage groups in the amphitetraploid genome of* Malus*. According to orthologous* bZIP* gene pairs analysis, the conserved and syntenic blocks were common to all three genomes analyzed, with a single syntenic block in peach corresponding to one or two syntenic regions in strawberry and two or four syntenic regions in apple. Vilanova et al. [62] compared the diploid reference linkage maps for* Prunus* and* Fragaria* and they identified numerous chromosomal translocations and rearrangements that occurred in the 29 million years since the genera diverged from a common ancestor. Notably,* bZIP* genes on the PC4 peach chromosome corresponded orthologously not to FC6, but rather to FC3. The data indicated that two genes (*ppa016271m* and* ppa022385m*) located on a nonorthologous chromosome region that had originated from a common ancestor went through some intrachromosomal rearrangements. This interpretation is consistent with the fact that a greater number of small-scale rearrangements occurred in strawberry in comparison to either apple or peach [42]. Whilst an early hypothesis as to the origin of* Malus* implied wide hybridization between ancestral amygdaloid (*x* = 8) and ancestral spiraeoid (*x* = 9) [63], other data suggest that* Malus* may have arisen due to polyploidization of a spiraeoid species [64]. Illa et al. [23] reconstructed a hypothetical ancestral genome for the Rosaceae containing nine chromosomes (*x* = 9), consistent with the report of Vilanova et al. [62]. Based on the analysis of orthologous pairs between chromosomes, we could propose a hypothesis that these orthologs became after one gene duplication located on one of the nine ancient chromosomes in the Rosaceae. An evaluation of the conservation of synteny between* Fragaria*,* Malus*, and* Prunus* based on whole genome sequence data may reveal much about sequence evolution in this closely related family.

## Supplementary Material

Figure S1. Phylogenetic tree constructed by the neighbor-joining method using protein sequences of the bZIP-domains in strawberry, apple, peach, and A. thaliana. Only bootstrap values larger than 50% are indicated. Different colors can be used to distinguish the different subgroups. The names of each subgroup are listed on the right.
Table S1. bZIP genes in C. sativus, M. truncatula, P. trichocarpa, B. rapa, C. papaya, T. cacao, S. lycopersicum, M. acuminata, S. moellendorffii, P. patens, C. reinhardtii, C. merolae, and S. cerevisiae.
Table S2. bZIP genes in strawberry, apple and peach. Gene name, chromosomal location, sequence and location of bZIP-domain, sequence of the full-length protein.
Table S3. Ka/Ks ratio of bZIP genes in clade A-S.
Table S4. Ka/Ks ratio of bZIP orthologs and paralogs in clades A-S.
Table S5. Ka/Ks ratios of bZIP domains, BR and LZ domains in clades A-S.
Table S6. Orthologous gene pairs of bZIP genes in strawberry, apple and peach. Gene name, clade, location, ORF length, and number of exons (Fv–strawberry; Md–apple; Pp–peach).
Table S7. Orthologous chromosomes in strawberry, apple and peach (FC–strawberry chromosome; MC–apple chromosome; PC–peach chromosome).
