# Supplementary material for: Divergence of the bZIP Gene Family in Strawberry, Peach, and Apple Suggests Multiple Modes of Gene Evolution after Duplication
Source: Int J Genomics. 2015 Dec 7;2015:536943. doi: 10.1155/2015/536943 (PMC4685131; doi:10.1155/2015/536943)
Supplement: Supplementary file 1 — Figure S1. Phylogenetic tree constructed by the neighbor-joining method using protein sequences of the bZIP-domains in strawberry, apple, peach, and A. thaliana. Only bootstrap values larger than 50% are indicated. Different colors can be used to distinguish the different subgroups. The names of each subgroup are listed on the right. Table S1. bZIP genes in C. sativus, M. truncatula, P. trichocarpa, B. rapa, C. papaya, T. cacao, S. lycopersicum, M. acuminata, S. moellendorffii, P. patens, C. reinhardtii, C. merolae, and S. cerevisiae. Table S2. bZIP genes in strawberry, apple and peach. Gene name, chromosomal location, sequence and location of bZIP-domain, sequence of the full-length protein. Table S3. Ka/Ks ratio of bZIP genes in clade A-S. Table S4. Ka/Ks ratio of bZIP orthologs and paralogs in clades A-S. Table S5. Ka/Ks ratios of bZIP domains, BR and LZ domains in clades A-S. Table S6. Orthologous gene pairs of bZIP genes in strawberry, apple and peach. Gene name, clade, location, ORF length, and number of exons (Fv–strawberry; Md–apple; Pp–peach). Table S7. Orthologous chromosomes in strawberry, apple and peach (FC–strawberry chromosome; MC–apple chromosome; PC–peach chromosome). [file 536943.f1.zip › 536943.f1/Supplementary/Supplementary Figure legends.docx]

**Figure S1. Phylogenetic tree constructed by the neighbor-joining method using protein sequences of the bZIP-domains in strawberry, apple, peach, and *A. thaliana*.** Only bootstrap values larger than 50% are indicated. Different colors can be used to distinguish the different subgroups. The names of each subgroup are listed on the right.
